# Evaluation of Antidiabetic and Antioxidant Properties of *Brucea javanica* Seed

**DOI:** 10.1155/2014/786130

**Published:** 2014-02-05

**Authors:** Abdulwali Ablat, Jamaludin Mohamad, Khalijah Awang, Jamil A. Shilpi, Aditya Arya

**Affiliations:** ^1^Institute of Biological Science, Faculty of Science, University of Malaya, 50603 Kuala Lumpur, Malaysia; ^2^Department of Chemistry, Faculty of Science, University of Malaya, 50603 Kuala Lumpur, Malaysia; ^3^Department of Pharmacy, Faculty of Medicine, University of Malaya, 50603 Kuala Lumpur, Malaysia

## Abstract

The ethanol extract of *B. javanica* seed was fractionated with solvents of different polarities and tested for antioxidant activities by several assays including DPPH radical scavenging activity, ferric reducing antioxidant power (FRAP), ferrous ion chelating activity (FCA), and nitric oxide radical scavenging activity (NORSA) along with their polyphenolic contents. Antidiabetic activity was evaluated both in vitro and in vivo using a glycogen phosphorylase **α** (GP**α**) inhibition assay and oral glucose tolerance test (OGTT) in nondiabetic rats. The ethyl acetate fraction (EAF), rich in tannin, exhibited the strongest antioxidant activities to DPPH, FRAP, and NORSA, except for FCA. The EAF also exerted a dose-depended inhibition of GP**α** (IC_50_ = 0.75 mg/ml). Further evaluation of hypoglycemic effect on OGGT indicated that rats treated with EAF (125 mg/kg bw) showed a 39.91% decrease (*P* < 0.05) in blood glucose levels at 30 min, and continuous fall (*P* < 0.05) of 28.89% and 20.29% was observed in the following hours (60 and 90 min) compared to the normal control during OGTT. The EAF was applied to polyamide column chromatography, and the resulting tannin-free fraction was tested for both GP**α** inhibition and antioxidant (DPPH only) activity. The GP**α** inhibitory activity was retained, while antioxidant activity was lost (4.6-fold) after tannin removal. These results concluded that the GP**α** inhibitory activity initially detected was primarily due to the compounds other than tannins, whereas antioxidant activity was mainly due to the tannins.

## 1. Introduction

Diabetes is one of the most common chronic diseases characterized by hyperglycemia as a result of impaired insulin secretion by pancreatic *β* cells and by cellular resistance to insulin [1]. Diabetes mellitus is recognized by the World Health Organization (WHO) as a tremendously increasing global epidemic with more than 285 million people around the world afflicted in 2010 and it is estimated that the number of people with diabetes will increase to 439 million by 2030 [2]. The current pharmacological treatment of diabetes is aimed at maintaining strict control of glycemia using oral hypoglycemic agent and insulin or combination of both. However, currently available oral antihyperglycemic agents, even when used intensively, are often unable to control the hyperglycemia and the disease progressively worsens with time. Oxidative stress has been suggested to be critically involved in the pathogenesis and progression of diabetes, including cardiovascular diseases [3], chronic kidney disease [4], ageing [5], and diabetes [6].

The ethnopharmacological evidence has proven that the use of herbal medicine is a viable alternative for the control of diabetes and other diseases. The beneficial properties of herbal medicine include significant efficiency, fewer side effects, relative safety, and especially low cost for a patient who cannot afford precious medication [7]. In fact, the medicinal plants are important natural sources of molecules with potential antidiabetic effects. Many plant species have been reported to have hypoglycemic effect, which may act through different mechanisms, including inhibition of *α*-glucosidase [8], inhibition of DPP-IV [9], and inhibition of glycogen phosphorylase and/or enhancement of insulin secretion, stimulation of glucose uptake [10]. The wide diversity of plant species has led scientists to make great efforts to bioprospect plants that may contribute to the control of diabetes.

*Brucea javanica* (*B. javanica*), a family of Simaroubaceae, is a 3 meter tall shrub which is mostly originated in India, Southeast Asia, and Northern Australia [11]. In traditional folk medicine, the seed of this plant has been used for the treatment of diabetes [12] and various disorders among indigenous peoples in Malaya peninsula.

Chemical compounds have been reported to be isolated from this plant including alkaloids [13], lignans and terpenoids [14], alkaloid glycosides [15], quassinoid glycosides [16], and quassinoids [17]. Quassinoids are known to be the major compounds isolated from Simaroubaceae family and they possess a variety of biological activities such as anticancer [18], antitumor [19]. It has been reported that quassinoid compounds bruceines E and D, isolated from butanol fraction of *B. javanica* seed, can reduce blood glucose concentration in short screening [12]. The aim of this study was, therefore, to investigate the hypoglycemic properties of *B. javanica* seed through different mechanisms using glycogen phosphorylase *α* (GP*α*) inhibition assay and to evaluate its antioxidant activities.

## 2. Materials and Methods

### 2.1. Chemicals and Reagents

Glycogen phosphorylase *α* from rabbit muscle, glycogen from rabbit liver (type III), *α*-D glucose-1-phosphate, HEPES [4-(2-hydroxyethyl) piperazine-1-ethanesulfonic acid, N-(2-hydroxyethyl) piperazine-N′-(2-ethanesulfonic acid)], magnesium chloride (MgCl2), EGTA (ethylene glycol-bis(2-aminoethylether)-N,N,N′,N′-tetraacetic acid), ammonium molybdate, malachite green, caffeine and potassium chloride, butylated hydroxyanisole (BHA), 2,2 diphenyl-1-picrylhydrazyl (DPPH), 2,4,6-tripyridyl-s-triazine (TPTZ), gallic acid monohydrate, ferrozine, sodium nitroferricyanide (III) dehydrate, sodium nitrite, Griess reagent, curcumin, polyvinyl polypyrrolidone (PVP), sodium acetate trihydrate, sodium phosphate dibase, sodium phosphate monobasic, and quercetin dihydrate were purchased from Sigma Chemical Co. (St. Louis, MO, USA). Ascorbic acid, acetic acid glacial, sodium chloride, ferrous sulfate (FeSO_4_), ferric chloride hexahydrate (FeCl_3_·6H_2_O), ethylenediaminetetraacetic acid disodium dehydrate (EDTA-Na_2_·2H_2_O), Folin-Ciocalteu phenol reagent, and sodium carbonate were purchased from Merck Chemical Co. (Malaysia). HPLC grade water was purchased from Fisher Scientific (Malaysia). All chemicals used are of analytical grade and were used without further purifications.

### 2.2. Sample Collection

Seeds from naturally grown* Brucea javanica* were collected from Tampin, Negeri Sembilan, Malaysia. The botanical identity of the sample was established based on morphology of the seeds and vegetative parts. The identity was confirmed by comparing with a voucher specimen available in the University of Malaya botanical garden. The seeds were dried in an oven at 40°C and stored in a moisture-free capped bottle at 2–8°C until use.

### 2.3. Preparation of Extracts

The powdered *Brucea javanica* seed (2 kg) was extracted three times with 95% ethanol (5 liter) at room temperature for three days. The extract was filtered with Whatman filter paper and combined together. The ethanol in the extract was removed by rotary evaporation. The residue was added to distilled water (200 mL × 3) to form a suspension and partitioned with n-hexane (250 mL × 3), chloroform (250 mL × 3), and ethyl acetate (250 mL × 3) to obtain a hexane (HF), chloroform (CHF), and ethyl acetate (EAF) soluble fractions. The residue was lyophilized to give as water fraction (WF). Each dried fraction was then tested for its biological activity.

### 2.4. Determination of Polyphenolic Contents in *B. javanica* Seed

#### 2.4.1. Determination of Total Phenolic Contents

Total phenol content (TPC) was measured by the Folin-Ciocalteu method [20]. Briefly, 20 *μ*L of sample extracts was mixed with 100 *μ*L of Folin-Ciocalteu reagent (diluted 10-fold with distilled water) in a 96-well microplate, incubated for 5 min, and 75 *μ*L of sodium carbonate solution (75 g/L) was added. After incubation period of 2 h in darkness at room temperature, the absorbance was measured at 740 nm with a microplate reader (Tecan Sunrise, Austria). Tannic acid (100–1000 *μ*M) was applied as standard for calibration and construction of a linear regression line and water was used as blank. The total phenolic content was estimated as mg tannic acid equivalent (mg TAE)/g of dry extract.

#### 2.4.2. Determination of Total Extractable Tannin Contents

Total extractable tannins (TET) were determined according to the method [21] with some modification. Sample solutions were prepared by dissolving 10 mg of samples in water (10 mL) for water fraction and methanol-H_2_O (1 : 1, 10 mL) for hexane, chloroform, and ethyl acetate fractions. The polyvinyl polypyrrolidone (PVP, 1.1 g) was added to the solutions, and the mixtures were vortexed thoroughly and centrifuged (3000 rpm, 15 min, 4°C) to precipitate the tannin. Phenolic contents in the supernatant which corresponds to the nonprecipitable phenol (NPP) were determined by the Folin-Ciocalteu method [20]. The TET was calculated as differences using the following equation: TET = TPC − NPP, and results were expressed in terms of mg tannic acid equivalent (mg TAE)/g of dry extract.

#### 2.4.3. Determination of Total Flavonoid Contents

Total flavonoid contents (TFC) were measured according to the method [22] with some modification. Briefly, extracts (50 *μ*L) were added with 70 *μ*L of distilled water and 15 *μ*L of 5% sodium nitrite solution in a 96-well microplate. The solutions were well mixed and incubated for 5 min at room temperature. Then, 15 *μ*L of 10% aluminum chloride solution was added into the mixture. After 6 min of incubation, 100 *μ*L of 1 M sodium hydroxide solution was added, and the absorbance was measured at 510 nm with a microplate reader (Tecan Sunrise, Austria). The total flavonoid contents were estimated from quercetin (200–1000 *μ*M) standard curve, and the results were expressed as mg quercetin equivalent (mg QE)/g of dry extract.

### 2.5. Antioxidant Activity Assays

#### 2.5.1. DPPH Radical-Scavenging Activity

The free radial scavenging activity of extracts was measured in terms of hydrogen donating ability using DPPH radical as described by the method [23] with a slight modification. Briefly, 40 *μ*L of sample extracts of different concentrations (0.05–2 mg/mL) were mixed with 200 *μ*L of 50 *μ*M DPPH solution in ethanol. The mixture was immediately shaken and incubated for 15 min in the dark at room temperature. The decrease in absorbance was measured at 517 nm with a microplate reader (Tecan Sunrise, Austria). BHA (5–80 *μ*g/mL) was used as a standard and the control was ethanol. The percentage of inhibition activity of the extracts was calculated according to the following equation:

(1) DPPH  radical  scavenging  activity  (%)  =Acontrol−Asample  or  standardAcontrol×100.

The concentration of extracts required to scavenge 50% of DPPH radical was estimated from the graph plotted against the percentage inhibition and compared with the standard. All the tests were performed in triplicate, and the results were expressed as *μ*g/mL.

#### 2.5.2. Ferric Reducing Antioxidant Power (FRAP)

The FRAP activities of extracts were measured as previously described method [20]. Twenty microliters of extracts in ethanol were mixed with 200 *μ*L of daily prepared FRAP reagent, which contained 5 mL 10 mM TPTZ in 40 mM HCl, 5 mL of 20 mM FeCl_3_, and 50 mL of 0.3 M acetate buffer (pH 3.6) in 96-well microplate. After 8 min of incubation time, the formation of the TPTZ-Fe^2+^ complex in the presence of antioxidant compounds in the extract was measured at 595 nm with a microplate reader (Tecan Sunrise, Austria). Ethanol was used as blank. Ferrous sulfate (FeSO_4_) solution (0.2 mM to 1 mM) was used for standard calibration curve. The FRAP value was evaluated according to the linear regression between standard solutions and absorbance at 595 nm and the results were estimated as mmol Fe^2+^/g of dry extract from triplicated tests.

#### 2.5.3. Metal Chelating Activity

The ferrous ion chelating activity (FCA) of the extracts was determined according to the procedure [24] by measuring the formation of the Fe^2+^-ferrozine complex based on the method as described by [25]. Extracts (100 *μ*L) at different concentrations (50–800 *μ*g/mL) were mixed with 120 *μ*L distilled water and 10 *μ*L FeCl_2_ (2 mM) in a 96-well microplate. Ferrozine (5 mM, 20 *μ*L) was added to the mixture to initiate the reaction. The reaction mixture was incubated at room temperature for 20 min and absorbance at 562 nm was measured along with EDTA-Na_2_ (5–80 *μ*g/mL) as a standard metal chelator. Ethanol (100 *μ*L) was used as a control; blank was without ferrozine (20 *μ*L of distilled water instead of ferrozine). The percent inhibition of Fe^2+^-ferrozine complex formation was calculated according to the following formula:

(2) Ferrous  ion  chelating  activity  (%)  =Acontrol−Asample  or  standardAcontrol×100.

The concentration of extracts required to chelate 50% of the Fe^2+^ ion (IC_50_) was calculated from the graph against the percentage of inhibition. All the tests were performed in triplicate, and the results were expressed as *μ*g/mL.

#### 2.5.4. Nitric Oxide Radical Scavenging Activity

Nitric oxide radical scavenging activity (NORSA) of fractions was determined according to the method [24] by measuring the formation of the nitrite ions in the reaction mixture that can be detected by Griess reagent. Fifty microliters of sample solutions at different concentrations (100−1600 *μ*g/mL) and an equal amount of sodium nitroferricyanide (10 mM) in phosphate-buffered saline (20 mM, pH 7.4) were mixed well in a 96-well microplate. The mixture was incubated at room temperature for 150 min and 125 *μ*L of Griess reagent was added. After 10 min, the absorbance was measured at 546 nm with a microplate reader (Tecan Sunrise, Austria). Curcumin (10–160 *μ*g/mL) and ethanol were used as a standard and control. The reaction mixture without Griess reagent was served as blank. The percent inhibition of nitric oxide was calculated using the formula:

(3) Nitric  oxide  radical  scavenging  activity  (%)  =Acontrol−Asample  or  standardAcontrol×100.

The concentration of extracts needed to scavenge 50% of the nitric oxide (IC_50_) was estimated from the graph against the percentage of inhibition. All the tests were performed in triplicate, and the results were expressed as *μ*g/mL.

### 2.6. Determination of Glycogen Phosphorylase Activity

Glycogen phosphorylase *α* activity of each fraction was measured in the direction of glycogen synthesis by the release of phosphate from glucose-1-phosphate as described by [26] with some modification. The sample or standard (10 *μ*L) was mixed with 40 *μ*L of 50 mM Hepes (pH 7.2) containing 100 mM KCl, 2.5 mM EGTA, 2.5 mM MgCl_2_, 0.25 mM glucose-1-phosphate, and 1 mg/mL glycogen in a microplate reader. The reaction was initiated by mixing 50 *μ*L of enzyme (GP*α*) in 50 mM Hepes (pH 7.2) buffer and incubated at 22°C for 30 min. The mixture was subsequently incubated with 150 *μ*L of 1 M HCl solution containing 10 mg/mL ammonium molybdate and 0.38 mg/mL malachite green for 5 min, and phosphate was measured at 620 nm. The caffeine was used as a standard and Hepes buffer (50 mM, pH 7.2) was used as a control. The inhibition percentage of glycogen phosphorylase activity was calculated using the following equation:

(4) GPα  inhibition  (%)  =Acontrol−Asample  or  standardAcontrol×100.

All the tests were performed in triplicate, and the results were expressed as mg/mL.

### 2.7. Polyamine Chromatography

Polyamide column chromatography was performed according to the method explained by [27], with some modifications. The extract (70 mg) was dissolved in mixture of ethyl acetate and methanol (1 : 1, 10 mL) and applied to column containing polyamide (2 g) pre-equilibrated with mixture of EA and MeOH. The column was eluted under the vacuum, and washed with a same solvent system until the elution was clear. The elution was dried and tested for GP*α* inhibition assay.

### 2.8. Experimental Animals

Sprague Dawley (SD) rats of both sexes weighing 200–250 g purchased from University of Malaya Animal Experimental Unit were used in this study. The rats were housed in cages under standard laboratory conditions with tap water and standard rat pallet diet, in a 12 h/12 h light/dark cycle at a temperature of 22 ± 1°C with a relative humidity of 65 ± 5%. The study was conducted in accordance with the principles outlined in the guide for “Animal Use Protocol” prepared by the University of Malaya Animal Care and Use Committee (Ethic no. ISB/23/05/2013/AA (R)).

### 2.9. Oral Acute Toxicity

The oral acute toxicity (OAT) of EA fraction from *B. javanica* seed was evaluated in healthy adult SD rats according to the guidelines of the Organization for Economic Cooperation and Development [28]. The rats were fasted overnight (16 h), divided into 6 groups (*n* = 6), and orally fed EAFs at the doses of 50, 125, 200, 250, and 300 mg/kg body weight. The control groups received distilled water only. Rats were observed continuously for 2 h, and then at 6 h intervals for 24 h and finally after every 24 h up to 14 days for any physical signs of toxicity such as food consumption, urination, writhing, gasping, response to touch, and decreased respiratory rate or for any lethality.

### 2.10. Oral Glucose Tolerance Test in Nondiabetic Rats

The SD rats (either sex) were divided into 4 groups (*n* = 6), fasted overnight (16 h), and fed with EAFs (50 or 125 mg/kg), glibenclamide (standard drug, 10 mg/kg) in a volume of 10 mL/kg bw by oral gavage, respectively. The control groups received distilled water. Glucose (2.0 g/kg bw) was fed to all groups 30 min after the treatment of EAF or standard. Blood glucose levels were measured at 0, 30, 60, 90, 120, and 180 minutes after glucose load using Accu-check glucose test strips and glucose meter (Accu-check, Roche Diagnostics, USA) [29]. Data are expressed as the mean ± standard error of the mean (SEM). The difference between the groups was statistically significant as determined by one-way ANOVA followed by Dunnett's post hoc multiple comparison test (*P* < 0.05) using the statistical program (SPSS 16.0 version, Chicago, IL, USA).

### 2.11. Statistical Analyses

Results were expressed as the mean ± standard error (SEM) for the three independent experiments. Differences between extracts were analyzed by one-way ANOVA followed by Dunnett's post hoc multiple comparison test at the 5% level (*P* < 0.05). The correlation between phenol, tannin, or flavonoid contents and antioxidant activity was estimated using Pearson's correlation test using Microsoft Excel. The statistical program (SPSS 16.0 version, Chicago, IL, USA) was performed in the entire test.

## 3. Results and Discussion

### 3.1. Extraction Yields

In the present study, the 95% ethanol was selected for extraction solvent, because it was less toxic, widely used in food, pharmaceutical, and cosmetic industry, and best suited for extracting a broad range of chemical compounds that may be found in the plant. In an attempt to separate the chemical compounds according to polarity, nonpolar to highly polar solvents were chosen. In the first step, *B. javanica* seed powder (2 kg) was extracted with 95% ethanol to give an ethanol extract (53.6 g). It was then suspended in distilled water and partitioned with n-hexane, chloroform, and ethyl acetate. The yields have varied depending on extraction solvents used, where water fraction (WF) gave 20.4 g of light brown solid with the highest extraction yield, followed by hexane (HF) which yielded 18.1 g of green oil. The chloroform fraction (CHF) gives a green powder with a medium yield (3.2 g), and the lowest extraction yield (1.7 g) was obtained from ethyl acetate fraction (EAF). In general, plant seeds contain proteins, amino acids, lipids, polysaccharides, alkaloids, polyphenols, flavonoids, and so on. In the form of mixture, mostly lipids can be found in nonpolar phase of solvent system, and proteins, amino acids, and other hydrophilic molecules such as polyphenols remained in polar phase [30]. Therefore, the differences in extraction yields could be explained by a higher mass transfer between solvents due to hydrophobic interactions with water phase.

### 3.2. Polyphenolic Contents of *B. javanica* Seed

The various contents of polyphenol in *B. javanica* seed were detected among the fractions, and the results are presented in Table 1. The results showed that, depending on the types of solvent used, the quantities of total phenol contents (TPC), total extractable tannins (TET), nonprecipitable phenol (NPP), and total flavonoid contents (TFC) were significantly different (*P* < 0.05) in each fraction. The highest amount of TPC (169.03 ± 3.54 mg TAE/g), TET (107.00 ± 1.74 mg TAE/g), and TFC (154.73 ± 0.61 mg QE/g) was detected in ethyl acetate fraction (EAF), and chloroform fraction (CHF) yielded TPC (119.98 ± 2.58 mg TAE/g), TET (15.02 ± 2.21 mg TAE/g), and TFC (49.45 ± 4.46 mg QE/g). The medium yields of TPC, TET, and TFC were observed in WF, where they were 46.71 ± 4.08 mg TAE/g, 11.71 ± 5.12 mg TAE/g, and 33.33 ± 5.77 mg QE/g, respectively. The lowest TPC (0.82 ± 0.39 mg TAE/g), TET (0.25 ± 1.50 mg TAE/g), and TFC (15.60 ± 4.82 mg QE/g) were detected in HF. When compared, polyphenol contents between organic and aqueous mediums, most of the polyphenol was extracted with organic solvent that has high polarity, and amount of polyphenol was increased with the increased polarity of the solvent. The WF yielded medium quantities of polyphenol content (Table 1). In agreement with this result, T. Wang et al. [31] reported earlier that polyphenolic compounds were best soluble in polar organic solvent than in water. In contrary, Y. Cai et al. [32] reported that TPC in water extract was higher than that in methanol extract of *B. javanica* seed collected from China. This difference could be due to the season and the location where the plant was collected, as well as solvents and extraction techniques applied.

### 3.3. Antioxidant Activity Assay

#### 3.3.1. DPPH Radical Scavenging Activity Assay

The present method to determine DPPH radical scavenging activity is based on the formation of the DPPH-H nonradical form in the presence of hydrogen donating antioxidants in the extracts that can be detected at 517 nm. The DPPH radical scavenging activities of *B. javanica* seed extracts were tested at different concentrations against the BHA as a standard. As shown in Table 1, the highest DPPH scavenging activity was observed in EAF (IC_50_ = 33.65 ± 3.04 *μ*g/mL) and it was approximately 5.5-fold lesser when compared to the BHA (IC_50_ = 5.95 *μ*g/mL). The WF (IC_50_ = 184.58 ± 7.31 *μ*g/mL) exhibited 31-fold less activities than that of standard, respectively. Hexane and chloroform fractions exhibited the lowest effect to reduce DPPH free radical, where maximal inhibitions were found 22.24% and 33.92% at the concentration tested (2 mg/mL). As noted in Tables 1 and 2, only the extracts in more polar organic solvent contained high level of TPC, TFC, and TET and exhibited potent efficiency to DPPH, suggesting that polyphenolic compounds may be the main contributor to scavenging DPPH free radicals in the *Brucea javanica* seed.

#### 3.3.2. Ferric Reducing Antioxidant Power (FRAP) Assay

The FRAP value of extracts was measured based on the reaction between antioxidant potentials and Fe^3+^-TPTZ complex to produce blue color Fe^2+^-TPTZ form in the extracts. In accordance with DPPH radical scavenging ability (Table 1), the higher FRAP value was detected in EAF (1.64 ± 0.06 mmol Fe^2+^/g of dry extracts) followed by CHF (0.24 ± 0.01 mmol Fe^2+^/g of dry extracts) in *Brucea javanica* seed. The reducing ability of hexane (0.14 ± 0.01 mmol Fe^2+^/g of dry extracts) was almost negligible where its value was found to be 0.14 ± 0.01 mmol Fe^2+^/g of dry extracts. The moderate reducing power was detected in the most polar solvent water extract (0.20 ± 0.01 mmol Fe^2+^/g of dry extracts). As mentioned earlier, just like DPPH, the extracts containing the high value of TFC, TPC, and TET showed the highest reducing power activity. The ferric reducing ability correlated well with TPC (*r* ^2^ = 0.9985), TFC (*r* ^2^ = 0.790), TET (*r* ^2^ = 0.997) contents in the fractions. Thus, it indicates that polyphenolic compounds are the most efficient in reducing power in *Brucea javanica* seed.

#### 3.3.3. Metal Chelating Activity

The assay performed to measure chelating ability of ferrous ion was based on the chelation of this ion with ferrozine to form ferrous-ferrozine complex which can be detected at 562 nm. The FCA of *B. javanica* seed fractions was measured at the different concentrations (100–800 *μ*g/mL) and the results are presented in Table 2. The results showed that HF (IC_50_ = 93.75 ± 7.75 *μ*g/mL) with significantly lowest polyphenol contents as well as weakest scavenging activities against DPPH and FRAP (Table 1) exhibited the highest ferrous ion chelating effects, whereas EAF (IC_50_ = 165.45 ± 5.65 *μ*g/mL) contained higher amount of polyphenol and has the highest efficiency to DPPH and FRAP and exhibited moderate ferrous ion chelating effects. The chloroform fraction containing high phenol (NPP) contents has shown inhibition with IC_50_ values of 314.55 ± 7.75 *μ*g/mL. The lowest inhibition was detected in the water fraction (19.72%) at a concentration of 800 *μ*g/mL. All the fractions showed low chelating activities when compared with EDTA-Na_2_ (IC_50_ = 10.47 ± 0.93 *μ*g/mL). The FCA of extracts correlated neither with TPC (*r* ^2^ = −0.177), TFC (*r* ^2^ = −0.297), TET (*r* ^2^ = −0.301), nor NPP (*r* ^2^ = 0.036) contents. Thus, these results indicate that polyphenolic compounds do not appear to be effective ferrous ion chelators in *B. javanica* seed.

#### 3.3.4. Nitric Oxide Radical Scavenging Activity Assay

The NORSA of the extracts was measured at different concentrations (100–1600 *μ*g/mL) and the results are shown in Table 1. The highest NO scavenging activity was observed in polyphenol-rich EAF with maximal IC_50_ value of 86.19 ± 3.83 *μ*g/mL) and it was approximately 9-fold lesser when compared with curcumin (IC_50_ = 9.50 ± 2.03 *μ*g/mL). The HF, CHF, and WF reached 36.04%, 16.08%, and 26.36% scavenging activities at a concentration of 1600 *μ*g/mL. As shown in Table 1 and Table 2, polyphenol-rich EAF again exhibited the strongest effect, suggesting that polyphenol is the main contributor to scavenging NO on *B. javanica* seed.

### 3.4. Glycogen Phosphorylase Inhibition Activity

**Figure 1:**
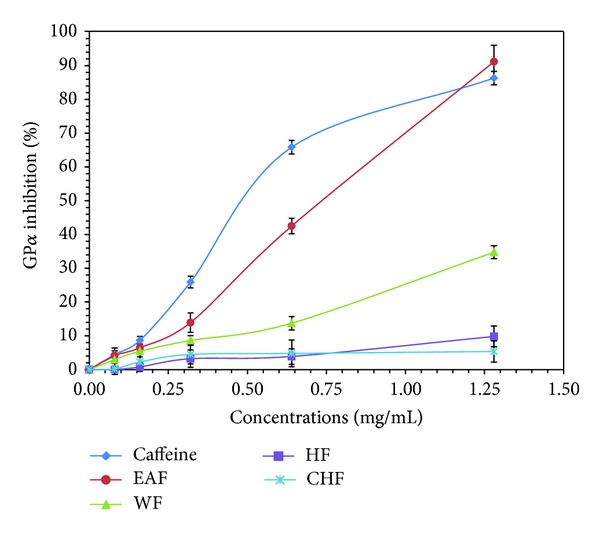
Glycogen phosphorylase *α* (GP*α*) inhibition by *B. javanica* seed. EAF: ethyl acetate fraction. WF: water fraction. HF: hexane fraction. CHF: chloroform fraction. The values are shown in mean ± SE (*n* = 3).

The GP*α* activity of *B. javanica* seed was evaluated against GP*α* from rabbit muscle in the direction of glycogen synthesis by the release of phosphate from glucose-1-phosphate. As shown in Figure 1, EAF inhibited the GP*α* activity in vitro in a dose-depended manner, reaching a 91.17% inhibition at the concentration of 32 mg/mL, and its IC_50_ value was 0.75 ± 2.30 mg/mL. This inhibition was 1.5-fold lesser compared to that of a standard (IC_50_ = 0.49 ± 2.00 mg/mL). The water fraction (WF) exhibited a slight efficiency towards GP*α*, where its maximal inhibition was found to be 34.75%. The poor GP*α* inhibitory activity was observed in HF (9.81%). The CHF, high in phenol (NPP) content, exhibited only 5.36% inhibition at a concentration of 32 mg/mL, suggesting that phenols (NPP) do not appear to be effective GP*α* inhibitors in *B. javanica *seed (Figure 1). The results (Figure 1) clearly identify that EA fraction exhibited the highest effect against GP*α* (Figure 1) than the other fractions studied here. In order to evaluate classes of compounds, which are responsible for GP*α* inhibitory and antioxidant activities, the most effective EAF was subjected to polyamide column chromatography to separate tannins from other compounds. On eluting the column with a mixture of EA-MeOH (1 : 1, 10 mL), nontannin contents were washed off from the column, while tannins were retained on the column. The EA-MeOH eluted fraction lost 24.13% of initial weight applied to the column, and tannins removed from the sample were confirmed as negative for tannins when the tannin test [33] was applied. If tannin-free EAF was tested against GP*α* and DPPH, the GP*α* activity was retained, whereas DPPH scavenging activity (IC_50_ = 155.39 *μ*g/mL) was lost 4.6-fold from its origin (IC_50_ = 33.65 *μ*g/mL) after tannin was removed. Therefore, it can be concluded that the GP*α* inhibitory activity initially detected in EAF was due to compounds other than tannins, whereas DPPH scavenging activity was due to the tannins.

### 3.5. Acute Toxicity

The OAT studies revealed that EAF was nontoxic nature at the dose of 50 and 125 mg/kg, and no lethality or any toxic effect was observed during 14 days of monitoring. Based on these findings, 50 mg/kg and 125 mg/kg doses were selected as maximum doses for further study.

### 3.6. Oral Glucose Tolerance Test in Nondiabetic Rats

**Figure 2:**
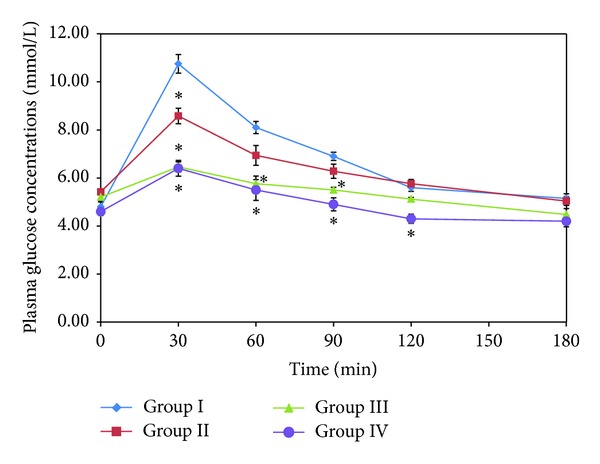
The effect of EAF from *B. javanica* seed on nondiabetic rats during the oral glucose tolerance test. The values are shown in mean ± SE (*n* = 6). Group I: normal control (distilled water); Group II: treated with EAF (50 mg/kg bw); Group III: treated with EAF (125 mg/kg bw); Group IV: treated with oral antidiabetic drug glibenclamide (10 mg/kg); all groups orally received glucose (2.0 g/kg bw) at 30 minutes after treatment. *P < 0.05 compared to the control at the corresponding time.

The hypoglycemic effects of EAF from *B. javanica* seed on the oral glucose tolerance test (OGTT) in nondiabetic rats are presented in Figure 2. The plasma glucose in the normal control group elevated its maximal level at the time point of 30 min after glucose (2.0 g/kg) loading and then gradually declined to initial level at 180 min. The treatment with a different doses (50 and 125 mg/kg) significantly (*P* < 0.05) reduced blood glucose levels by 20.19% and 39.91% at 30 min, and significant (*P* < 0.05) reduction in the following hours (at 60 and 90 min) was only observed at high dose (EAF, 125 mg/kg) treated rats compared to normal control. Rats treated with oral antidiabetic drug glibenclamide exhibited significant (*P* < 0.05) reduction in blood glucose levels at 30, 60, 90, and 120 min after glucose load compared to normal control (Figure 2). Finally, no difference was found between groups at 180 min.

## 4. Conclusion

The results from the current experiment showed that fractions of *B. javanica* seed contained various amounts of TFC, TPC, TET, and phenol (NPP) and greatly influenced their antioxidant properties. Most polyphenolic compounds were extracted with ethyl acetate and exhibited the highest activities to DPPH, FRAP, and NORSA, except for FCA (Table 2), suggesting that polyphenol is the main contributor for antioxidant activities of the extracts. Interestingly, HF containing significantly (*P* < 0.05) lowest polyphenol contents (Table 1) exhibited high value of ferrous ion chelating effects, and no correlation was found between polyphenolic contents and chelating activity, indicating that polyphenolic compounds do not appear to be effective ferrous ion chelators in *B. javanica* seed. All the fractions were tested for their antidiabetic activity using a GP*α* inhibition assay in vitro. The highest inhibition was again observed in EAF, showing an IC_50_ of 0.75 mg/mL in a dose-dependent manner, and therefore it was selected for further studies in our laboratory. In order to identify a correlation between antioxidant compounds and GP*α* inhibitors in *B. javanica* seed, the EAF was applied to polyamide column chromatography to separate tannins and was tested for both GP*α* inhibitory and antioxidant (DPPH only) activities. The GP*α* inhibitory activity initially detected remained unchanged, while antioxidant activity was lost 4.6-fold from its origin after tannin was removed, and no clear correlation was found between GP*α* and DPPH radical scavenging activity, indicating that tannins are not the main contributors for GP*α* inhibitory activity, but they are mainly responsible for the scavenging effects in this plant. The hypoglycemic effect of EAF was further evaluated by oral glucose tolerance test (OGTT) in nondiabetic rats. Along with GP*α* inhibitory activity, EAF effectively improved glucose tolerance (Figure 2) such as what standard antidiabetic drug glibenclamide did. Thus, these results offer scientific evidence for the use of *B. javanica* seed in the indigenous system.

## Figures and Tables

**Table 1:** Total phenolic and different classes of polyphenol contents in *B. javanica* seed.

Fractions	TFC (mg QE/g)	TPC (mg TAE/g)	TET (mg TAE/g)	NPP (mg TAE/g)
HF	15.60 ± 4.82^a^	0.82 ± 0.39^a^	0.45 ± 1.50^a^	0.37 ± 2.21^a^
CHF	49.45 ± 4.46^b^	119.98 ± 2.58^b^	15.02 ± 2.21^b^	104.97 ± 4.72^b^
EAF	154.73 ± 0.61^c^	169.03 ± 3.54^c^	107.00 ± 1.74^c^	62.02 ± 1.50^c^
WF	33.33 ± 5.77^d^	46.71 ± 4.08^d^	11.71 ± 5.12^d^	34.99 ± 1.75^d^

Data are mean ± SE (*n* = 3). The means with different lower case letters (a, b, c, and d) in the same column are significantly different at *P* < 0.05 (ANOVA, followed by Duncan's multiple comparison test). TFC: total flavonoid content expressed as mg quercetin equivalent (mg QE)/g of dry extract. TPC: total phenolic content; TET: total extractable tannin; NPP: nonprecipitable phenol contents expressed as mg tannic acid equivalent (mg TAE)/g of dry extract. HF: n-hexane fraction. CHF: chloroform fraction. EAF: ethyl acetate fraction. WF: water fraction.

**Table 2:** Antioxidant activities of *B. javanica* seed.

Fractions	DPPH (IC_50_ µg/mL)	FRAP (mmol Fe^2+^/g extract)	FCA (IC_50_ µg/mL)	NORSA (IC_50_ µg/mL)
HF	ND	0.14 ± 0.01^a^	93.75 ± 7.75^a^	ND
CHF	ND	0.24 ± 0.01^b^	314.55 ± 5.32^b^	ND
EAF	33.65 ± 3.04^a^	1.64 ± 0.06^c^	165.45 ± 5.65^c^	86.19 ± 3.83
WF	184.58 ± 7.31^b^	0.20 ± 0.01*	ND	ND

Data are mean ± SE (*n* = 3). Means with different lower case letters (a, b, c, and d) in the same column are significantly different at *P* < 0.05 (ANOVA, followed by Dunnett's multiple comparison test). *P < 0.05 compared to a and c in the same column. DPPH: 2,2 diphenyl-1-picrylhydrazyl. FRAP: ferric reducing antioxidant power. FCA: ferrous ion chelating activity. NORSA: nitric oxide radical scavenging activity. HF: n-hexane fraction. CHF: chloroform fraction. EAF: ethyl acetate fraction. WF: water fraction. ND: not detected.
